# Determination of Differential miRNA Expression Profile in People with Noise-Induced Hearing Loss

**DOI:** 10.3390/ijms26146623

**Published:** 2025-07-10

**Authors:** Gözde Öztan, Halim İşsever, Özlem Kar Kurt, Sevgi Canbaz, Fatma Oğuz, Tuğçe İşsever, Özmen Öztürk

**Affiliations:** 1 Department of Medical Biology, Istanbul Faculty of Medicine, Istanbul University, Topkapı, 34093 Istanbul, Turkey; oguzsf@istanbul.edu.tr; 2 Department of Public Health, Istanbul Faculty of Medicine, Istanbul University, Topkapı, 34093 Istanbul, Turkey; hissever@istanbul.edu.tr (H.İ.); sevgi.canbaz@istanbul.edu.tr (S.C.); 3 Yedikule Chest Diseases and Chest Surgery Training and Research Hospital, Zeytinburnu, 34020 Istanbul, Turkey; okarkurt@gmail.com; 4 Turkish Health Institutes Presidency (TUSEB), 34718 Istanbul, Turkey; tugceissever@gmail.com; 5 Acıbadem Kartal Hospital Ear, Nose and Throat Polyclinic, Kartal, 34873 Istanbul, Turkey; ozmen.ozturk@acibadem.com

**Keywords:** noise-induced hearing loss, miRNA array, pathogenesis, miRNAs

## Abstract

Noise-induced hearing loss (NIHL) is a significant occupational health issue, characterized by permanent damage to the cochlea due to prolonged exposure to high-intensity noise. Circulating microRNAs (c-miRNAs) have emerged as promising non-invasive indicators of inner ear pathology and potential modulators of cellular stress responses. Nevertheless, their specific roles in NIHL remain inadequately characterized. This study evaluated miRNA expression in the peripheral blood of individuals with bilateral NIHL (*n* = 12) and matched healthy controls (*n* = 6) using GeneChip^®^ miRNA 4.0 arrays. The Transcriptome Analysis Console software was used for differential expression analysis, and bioinformatic predictions of gene targets and pathway enrichment were performed using TargetScan (version 8.0) and the Enrichr tool. Among the 72 differentially expressed miRNAs (FDR < 0.05), hsa-miR-486-2, hsa-miR-664b-3p, hsa-miR-4485, hsa-miR-501, and hsa-miR-663b were notably upregulated, while hsa-miR-6723, hsa-miR-194-2, hsa-miR-668-5p, hsa-miR-4722-3p, and hsa-miR-4716 showed significant downregulation. Enrichment analyses indicated involvement in apoptosis regulation, mitochondrial stability, and cell cycle control. Principal component analysis (PCA) and clustering methods revealed clear molecular distinctions between the patient and control groups. The observed alterations in c-miRNA profiles highlight their relevance to NIHL-related cellular stress and degeneration. These findings support their utility as candidate biomarkers for diagnosis and prognosis, warranting further validation in functional and longitudinal studies.

## 1. Introduction

### 1.1. Noise-Induced Hearing Loss (NIHL) and Its Clinical Importance

Noise-induced hearing loss (NIHL) is recognized as a major occupational and environmental health problem worldwide. Chronic exposure to excessive noise can cause irreversible damage to the inner ear structures, resulting in permanent hearing impairment that affects millions of individuals globally [1]. Sensorineural hearing loss (SNHL) is among the most prevalent sensory deficits globally, typically arising from the damage or loss of cochlear hair cells and neurons that transmit auditory signals. While this condition is primarily linked to peripheral cochlear degeneration, it is increasingly recognized that central auditory pathways in the brain can also independently contribute to SNHL [2]. Clinically, NIHL often presents as a progressive, bilateral high-frequency hearing deficit caused by chronic exposure to damaging noise. Emerging evidence highlights that, beyond hair cell and spiral ganglion neuron loss, synaptic connections between inner hair cells and auditory nerve fibers are especially vulnerable, representing a key pathology in cochlear synaptopathy [3]. Occupational hearing loss is recognized as a permanent yet entirely preventable condition when appropriate prevention strategies and technologies are applied. Consistent prevention efforts, early detection, and timely intervention are critical for reducing the burden of hearing impairment among workers exposed to hazardous noise [4]. Effective prevention requires the regular monitoring of noise exposure levels and the use of engineering or administrative controls to reduce workplace noise. Where noise cannot be adequately reduced, providing appropriate personal hearing protective devices and training workers in their correct use are essential strategies [5].

Emerging evidence suggests that NIHL can present asymmetrically in some individuals, which may complicate differential diagnosis and appropriate clinical management [6]. Furthermore, novel insights into the underlying pathophysiology reveal that not only hair cells but also supporting cells and cochlear synaptic structures are vulnerable to noise-induced damage, suggesting broader targets for therapeutic intervention [7].

### 1.2. Molecular Mechanisms in Cochlear Injury

Cochlear injury remains a major contributor to hearing loss, and insights into its molecular mechanisms have expanded beyond mechanical trauma to include oxidative stress, metabolic dysfunction, and apoptotic pathways. This shift in understanding has informed the design of otoprotective strategies and clinical trials aimed at preventing both NIHL and drug-induced ototoxicity, given their shared cellular mechanisms [8]. The cochlea is highly sensitive to various insults including noise exposure, ototoxic drugs, aging, and genetic mutations. Emerging evidence implicates endoplasmic reticulum stress as a key driver of cochlear cell death in sensorineural hearing loss. The activation of the unfolded protein response, particularly via PERK, IRE1α, and ATF6 pathways, leads to oxidative stress, inflammation, and apoptosis in hair cells and spiral ganglion neurons, contributing to cochlear degeneration [9].

In particular, noise exposure induces the excessive production of reactive oxygen species (ROS), which contribute significantly to hair cell death through oxidative damage to cellular membranes, proteins, and DNA. Both apoptotic and necrotic pathways are triggered in the cochlea, with ROS acting as key mediators in this process. Consequently, antioxidant-based strategies have been explored as potential interventions to limit or reverse noise-induced cochlear damage [10]. Interestingly, oxidative damage in the cochlea may continue to develop even after the noise exposure has ceased, suggesting a prolonged pathological process. This delayed injury has been linked to the sustained production of reactive oxygen and nitrogen species, which peaks several days post-exposure and contributes to progressive hair cell degeneration. Such findings imply a potential therapeutic window during which antioxidant interventions could effectively mitigate further cochlear damage [11].

Recent findings challenge the traditional view of the cochlea as an immune-privileged organ, highlighting the presence of resident macrophages and perivascular immune cells in the lateral wall. These cells become activated in response to insults such as noise exposure, initiating local inflammatory responses. Proinflammatory cytokines, including TNF-α, IL-1β, and IL-6, are produced by cochlear fibrocytes and contribute to immune cell recruitment and tissue remodeling. Such immune activation presents potential targets for therapeutic intervention in SNHL [12]. Experimental evidence from avian models shows that caspase activation is a central mediator of both ototoxic and spontaneous hair cell death. The pharmacological inhibition of caspases significantly enhances hair cell survival following aminoglycoside exposure, suggesting a conserved apoptotic mechanism. Moreover, these findings imply that caspase-targeted therapies could be beneficial in preventing or reducing sensory hair cell loss [13].

Mitochondrial dysfunction contributes to cochlear damage through the loss of membrane potential, release of pro-apoptotic factors like cytochrome c, and increased oxidative stress. These mechanisms highlight the complex interplay between mitochondrial pathways, ROS generation, and intrinsic apoptotic signaling in sensory hair cell degeneration [14].

Although outer and inner hair cells may initially survive the acoustic insult, synaptic disconnection between inner hair cells and cochlear nerve fibers occurs rapidly after exposure, particularly in the high-frequency regions. This primary neural degeneration is not detected by standard threshold measurements, leading to an underestimation of the extent of injury. Studies using confocal imaging have revealed that, even after the full recovery of auditory thresholds, synaptic ribbon loss and spiral ganglion neuron degeneration may continue progressively over months. These findings redefine our understanding of “temporary” noise-induced hearing loss by highlighting its potential to trigger long-term and irreversible neural damage in the absence of overt hair cell loss [15].

### 1.3. Role of MicroRNAs (miRNAs) in Cochlear Pathology

MicroRNAs (miRNAs) are small non-coding RNAs that regulate gene expression post-transcriptionally by binding to complementary sequences in target mRNAs. In the cochlea, miRNAs are involved in maintaining cellular homeostasis and modulating responses to stress, injury, and aging processes [16]. The dysregulation of specific miRNAs has been shown to increase the vulnerability of cochlear hair cells and spiral ganglion neurons, contributing to sensorineural hearing loss. Notably, point mutations in the seed region of miR-96—a miRNA expressed in inner ear hair cells—have been causally linked to autosomal dominant, progressive non-syndromic hearing loss by impairing target recognition and miRNA biogenesis [17]. Noise-induced cochlear damage, even when auditory thresholds recover, can lead to lasting neural deficits not detected by standard hearing tests. Such exposures cause the rapid degeneration of afferent synapses beneath inner hair cells and the delayed loss of spiral ganglion neurons, redefining “temporary” NIHL as a potential trigger for irreversible neuropathy [15].

Several miRNAs, including miR-15a and miR-18a, have been identified as the critical regulators of hair cell development and maintenance in the inner ear. The conditional deletion of Dicer1, essential for miRNA processing, leads to the progressive degeneration of cochlear hair cells and ultimately results in profound hearing loss [18]. Mutations within the seed region of miR-96 have been directly linked to autosomal dominant, progressive hearing loss in humans. These alterations disrupt the miRNA’s ability to recognize and repress its target mRNAs, impairing the maintenance of gene expression essential for hair cell function. Functional studies demonstrate that such mutations not only reduce miRNA processing efficiency but also diminish the silencing of key auditory genes [17].

The miR-183 family, which includes miR-96, miR-182, and miR-183, is highly conserved across species and shows specific expression in mechanosensory hair cells. Functional studies in zebrafish have demonstrated that the overexpression of miR-96 or miR-182 leads to ectopic sensory structures, while their knockdown results in reduced hair cell numbers and morphological abnormalities. In humans, this miRNA cluster is located within a genomic region linked to autosomal dominant non-syndromic hearing loss. Such findings underscore the essential role of these miRNAs in inner ear development and suggest that their dysregulation may contribute to cochlear pathology [19]. Beyond gene silencing, miRNAs contribute to epigenetic regulation during inner ear development, influencing pathways that guide hair cell differentiation. Deviations in their expression can disrupt cochlear development and neuronal patterning, contributing to the various forms of hearing loss [20].

Changes in miRNA expression have been observed in the various forms of hearing loss, yet the upstream signals driving these alterations remain incompletely understood. Experimental findings suggest that ROS and pro-inflammatory cytokines may modulate miRNA profiles, thereby contributing to cochlear cell damage. Antioxidants, by modulating the expression of specific miRNAs, may offer a dual protective mechanism against both oxidative and inflammatory insults in the inner ear [21]. In the aging cochlea, the increased expression of miR-34a has been shown to suppress the SIRT1 levels, leading to the enhanced acetylation of the pro-apoptotic protein p53. This molecular cascade promotes outer hair cell apoptosis, particularly in the basal turn of the cochlea, where degeneration is most prominent. Interestingly, interventions such as resveratrol, a known SIRT1 activator, have demonstrated partial protection against miR-34a-induced hair cell loss and functional hearing decline [22].

### 1.4. miRNA Profiling in NIHL

MiRNA profiling has become an important tool in understanding the molecular changes associated with idiopathic sudden SNHL. C-miRNAs provide a minimally invasive means of assessing inner ear pathology, especially when direct tissue access is limited. Studies have shown that the expression levels of specific miRNAs, such as miR-183, miR-210, miR-23a, and miR-18b, differ significantly between patients with sudden SNHL and healthy controls. These circulating miRNAs have demonstrated potential as both diagnostic and prognostic biomarkers, reflecting underlying cellular damage and treatment response in this auditory disorder [23]. The miR-183 family, including miR-183, miR-96, and miR-182, shows highly conserved and robust expression in sensory hair cells of the inner ear. Alterations in the expression of these miRNAs have been linked to hair cell dysfunction and progressive hearing loss in animal models and humans [24].

Recent studies have demonstrated that miRNA expression profiles in perilymph correlate with auditory performance after cochlear implantation. Specific miRNAs identified in human inner ear fluids are associated with neuronal survival, inflammation, and metabolic pathways critical to hearing function. These findings suggest that miRNA dysregulation may reflect ongoing cochlear pathology and could serve as a predictor of long-term auditory outcomes. Integrating such miRNA signatures into clinical practice may aid in the early identification of individuals at risk for poor recovery or progressive hearing decline [25]. Short-term noise exposure can lead to significant alterations in the miRNA levels within the cochlear nucleus and inferior colliculus. Specific miRNAs, such as miR-183-5p, miR-411-3p, and miR-200b-3p, are thought to act as the key regulators of neural plasticity processes in the central auditory pathway. By targeting signaling cascades like MAPK and TGF-β, these miRNAs may offer promising molecular targets for developing therapeutic strategies against NIHL [26].

Growing evidence highlights that miRNAs play a significant role in inner ear development and the differentiation of auditory cells, reinforcing their relevance in hearing-related disorders. The dysregulation of specific miRNA expression has been linked to structural abnormalities in the cochlea and the loss of hair cell integrity, both of which contribute to sensorineural hearing loss. These observations emphasize the potential of miRNAs not only as indicators of cochlear injury but also as promising molecular targets for therapeutic approaches aimed at preserving or restoring hearing function [27]. The miR-183/96/182 cluster is highly expressed in sensory organs, including the inner ear, and is essential for maintaining normal auditory function. Subtle variations in their seed sequences enable both the cooperative and distinct regulation of gene networks, especially those related to inflammation and cellular stress responses. These features make the miR-183C cluster a compelling target for future therapeutic approaches aimed at mitigating damage and preserving sensory organ integrity [28].

### 1.5. Study Objective

This study aims to characterize the differential expression patterns of miRNAs and small RNAs in individuals diagnosed with bilateral sensorineural hearing loss due to occupational noise exposure. By identifying the key molecular alterations, we aim to expand the understanding of NIHL pathogenesis and propose novel biomarker candidates for diagnosis intervention.

## 2. Results

### 2.1. Sociodemographic and Audiometric Data in Noise-Induced Hearing Loss

The mean age of participants in the NIHL group was 47.75 ± 3.76 years, while that of the control group was 44.33 ± 4.13 years; this difference was not statistically significant (t = 1.75, *p* = 0.09). The mean duration of occupational exposure was 18.18 ± 5.67 years for the NIHL group and 16.33 ± 2.06 years for controls, with no significant difference observed (t = 0.75, *p* = 0.46). Thus, both groups were comparable in terms of age and work duration.

Among the 12 participants with bilateral noise-induced hearing loss, occupations included 4 foundry lathe operators, 2 textile workers, 2 installation technicians, 1 carpenter, 1 marble worker, 1 auto mechanic, and 1 road maintenance worker. In contrast, the control group consisted of six office workers not occupationally exposed to noise.

Regarding comorbidities, four patients in the NIHL group had asthma, and one patient had a confirmed diagnosis of migraine. Seven participants were determined to have no additional diseases. No comorbid conditions were reported in the control group.

Table 1 presents the pure-tone hearing thresholds across multiple frequencies. Statistically significant threshold shifts were observed in the NIHL group compared to controls at key frequencies in both ears. Specifically, right ear thresholds were significantly elevated at 1, 2, 4, 6, and 8 kHz (*p* < 0.05), while left ear thresholds were significantly elevated at 0.5, 2, 4, 6, and 8 kHz (*p* < 0.05). These shifts reflect the classic “acoustic notch” pattern typically observed in NIHL, particularly at 4 and 6 kHz, where hearing loss exceeded 50 dB on average.

### 2.2. Analysis of Differentially Expressed miRNAs and Small RNAs Using Transcriptome Analysis Console

Differential expression analysis was conducted using Transcriptome Analysis Console (TAC) version 4.0.3.14, based on data obtained from the GeneChip^®^ miRNA 4.0 Array (Affymetrix, Thermo Fisher Scientific, Santa Clara, CA, USA). The analysis was performed using the unpaired Student’s *t*-test, which compares the average expression levels (log2-transformed) between the independent patient and control groups to determine statistically significant differences. A volcano plot was generated to visualize the relationship between the magnitude of expression changes (log2 fold change) and their statistical significance (−log10 *p*-value). To correct for multiple testing, false discovery rate (FDR) adjustments were applied using the Benjamini–Hochberg procedure. Transcripts with a *p*-value less than 0.05 and a fold change of ≥2 or ≤−2 were considered significantly differentially expressed. Based on these criteria, 72 transcripts showed significant differential expression between the patient and control groups (Table 2).

**Figure 1:**
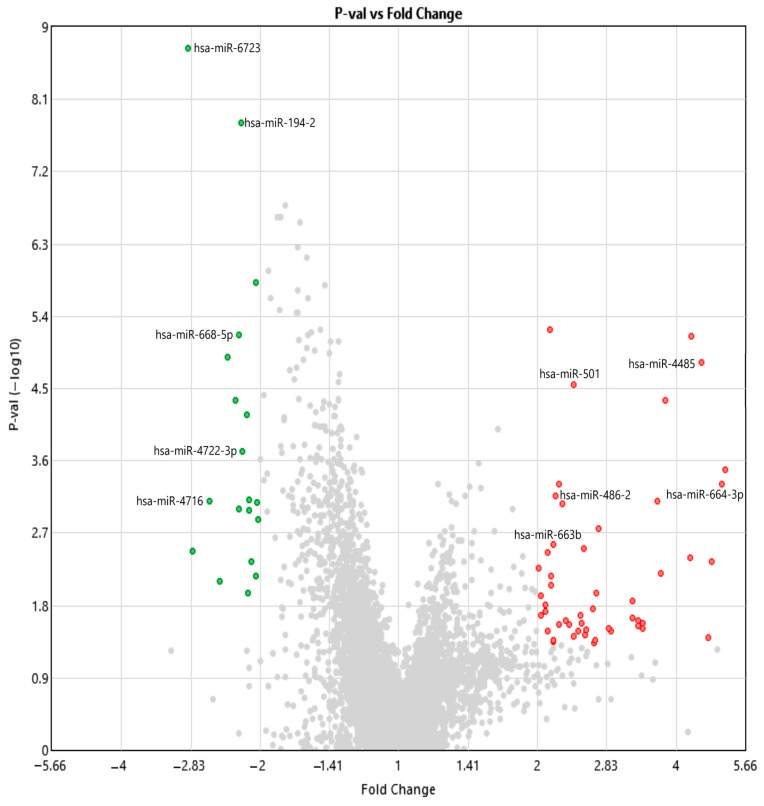
The volcano plot representing differentially expressed miRNAs and small RNAs obtained from the GeneChip^®^ miRNA 4.0 Array data, analyzed using the Transcriptome Analysis Console software version 4.0.3.14. The x-axis displays the log2 fold change (log2FC) in expression, calculated as patient vs. control, where negative values indicate downregulation in patients and positive values indicate upregulation. The y-axis shows the −log10 of the *p*-value, reflecting the statistical significance of the differential expression. Each dot represents an individual transcript (miRNA or small RNA). Red dots correspond to significantly upregulated transcripts in the patient group (log2FC > +2 and *p* < 0.05), while green dots denote significantly downregulated transcripts (log2FC < −2 and *p* < 0.05). Gray dots indicate transcripts that did not meet the defined thresholds for statistical significance. Transcripts farther from the center along the x-axis exhibit greater fold changes, while those higher on the y-axis are more statistically significant. Labeled points indicate the selected miRNAs of particular biological or diagnostic interest, primarily those identified as potential biomarker candidates in Table 4.

Out of the total differentially expressed transcripts, a selected subset of miRNAs surpassed the stringent thresholds (fold change ≤ −2 or ≥2 and *p* < 0.05), and this subset was identified as potential biomarker candidates, as summarized in Table 3. Five miRNAs (hsa-miR-6723, hsa-miR-194-2, hsa-miR-668-5p, hsa-miR-4722-3p, and hsa-miR-4716) exhibited significantly reduced expression in NIHL patients, whereas five others (hsa-miR-486-2, hsa-miR-664b-3p, hsa-miR-4485, hsa-miR-501, and hsa-miR-663b) were markedly upregulated. These miRNAs were also visualized in Figure 1 to emphasize their distinct expression patterns. In addition to these miRNAs, the transcriptome data revealed the differential expression of various small non-coding RNAs, including snoRNAs, scaRNAs, and snRNAs, indicating widespread disruptions in post-transcriptional regulatory mechanisms. These findings, further explored in Section 2.4 through pathway enrichment analyses, underscore the molecular heterogeneity of NIHL and lay the groundwork for future studies focused on regulatory network reconstruction and biomarker development.

### 2.3. miRNA Profiling Results Based on Hierarchical Clustering and Principal Component Analysis (PCA) Mapping

**Figure 2:**
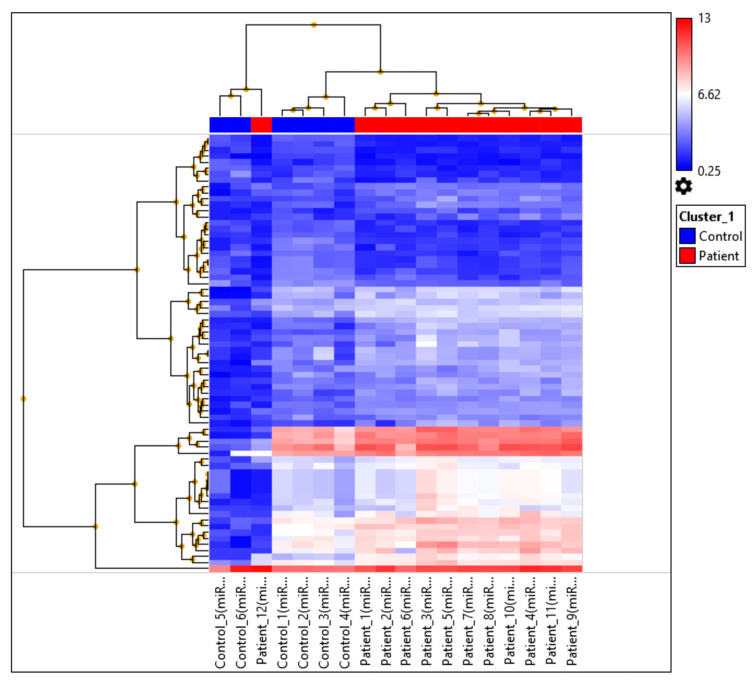
The hierarchical clustering and heatmap of miRNA expression profiles. Patient samples (red) and control samples (blue) are displayed. The clustering analysis revealed a clear separation between patients and controls, with Patient_12 clustering closer to the control group, suggesting individual variability within the patient cohort. Color intensity reflects relative expression levels, with red indicating higher expression and blue indicating lower expression.

Hierarchical clustering analysis was conducted to evaluate the similarities and differences in miRNA expression profiles between patients diagnosed with bilateral sensorineural hearing loss and healthy controls. The resulting heatmap and dendrogram (Figure 2) revealed a distinct separation between the two groups based on their expression patterns. Patient samples predominantly clustered together, indicating shared expression signatures, whereas control samples formed a separate, cohesive cluster.

Notably, while most patient samples grouped closely together, Patient_12 deviated from the main patient cluster and was positioned closer to the control group. This suggests that the miRNA expression profile of Patient_12 may differ from the typical molecular pattern observed in the patient cohort and may resemble the expression profile of healthy individuals. Overall, hierarchical clustering demonstrated that bilateral sensorineural hearing loss is associated with distinct miRNA expression changes that differentiate affected patients from healthy controls.

**Figure 3:**
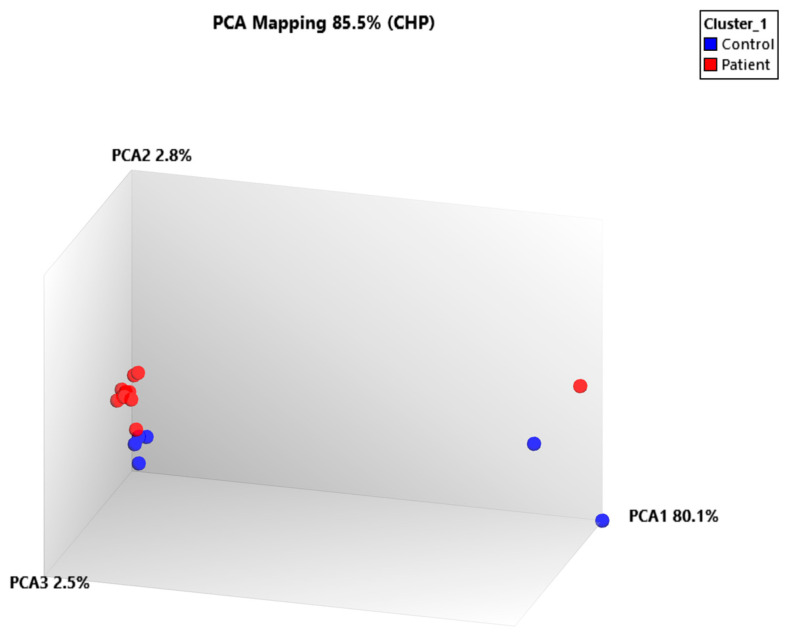
PCA mapping of microRNA expression profiles. Patient samples (red) and control samples (blue) are shown. Patient_12 was positioned closer to the control group, suggesting a molecular profile distinct from other patients. PCA1, PCA2, and PCA3 accounted for 85.5% of the total variance.

Principal component analysis (PCA) (Figure 3) showed a clear separation between the patient and control groups based on their miRNA expression profiles. The first three principal components (PCA1: 80.1%, PCA2: 2.8%, PCA3: 2.5%) collectively explained 85.5% of the total variance, indicating that the main sources of variation were robustly captured. Most patient samples clustered tightly together, whereas control samples formed a distinct and compact group. Notably, Patient_12 deviated from the main patient cluster and appeared closer to the control group, consistent with the hierarchical clustering results.

Collectively, these findings confirm that bilateral SNHL is associated with a distinct miRNA expression signature that clearly distinguishes affected individuals from healthy controls. The deviation observed in Patient_12 underscores the potential heterogeneity within the patient cohort, warranting further investigation into individual variability in disease-related molecular alterations.

To further interpret the molecular components underlying this separation, PC2 loading values were examined. The top contributing miRNAs with the highest absolute PC2 loadings were identified, highlighting transcripts most influential in distinguishing NIHL from control samples. Notably, miRNAs such as hsa-miR-486-2, hsa-miR-4722-3p, and hsa-miR-194-2 showed strong contributions to PC2 variance, reflecting their distinct up- or downregulated expression patterns. This analysis supports the notion that separation along PC2 is biologically meaningful and related to specific miRNA expression signatures (Table 3).

### 2.4. Pathway Enrichment and Biomarker Candidate Analysis

To identify the biological significance of the differentially expressed miRNAs, functional enrichment and target prediction analyses were conducted. Based on their low FDR, significant fold changes, and strong *p*-values, the following 10 miRNAs were selected as the most promising biomarker candidates: hsa-miR-6723, hsa-miR-194-2, hsa-miR-668-5p, hsa-miR-4722-3p, and hsa-miR-4716 (downregulated), and hsa-miR-4485, hsa-miR-501, hsa-miR-664b-3p, hsa-miR-486-2, and hsa-miR-663b (upregulated) (Table 4).

The strong statistical parameters and regulatory function of these miRNAs underline their potential utility as circulating biomarkers. Their presence in peripheral blood and association with well-defined biological pathways further support their relevance as diagnostic and potentially prognostic tools for NIHL. These results provide a foundation for future mechanistic and validation studies of these miRNAs as molecular biomarkers of noise-induced cochlear injury.

### 2.5. Distribution of Predicted miRNA–Target Binding Strengths

To evaluate the predicted binding strengths between differentially expressed miRNAs and their target genes, cumulative weighted context++ scores were obtained from the TargetScan 8.0 database. For each miRNA, the top 10 predicted target genes with the most negative context++ scores—representing the highest predicted binding affinity—were selected and compiled into a combined dataset.

**Figure 4:**
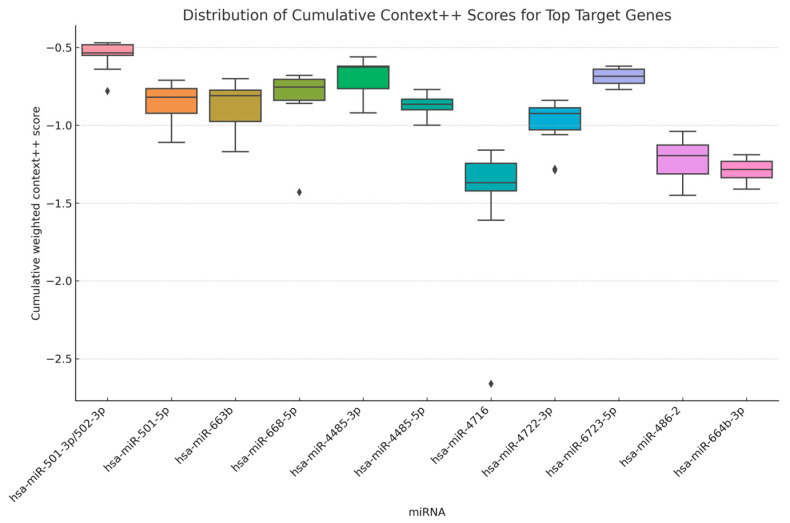
The boxplot showing the distribution of cumulative weighted context++ scores for the top 10 predicted target genes of each miRNA.

A boxplot was generated to visualize the distribution of these scores across all analyzed miRNAs (Figure 4). The plot revealed marked variation in score distribution among different miRNAs. For example, hsa-miR-486-2 and hsa-miR-664b-3p showed a relatively narrow interquartile range (IQR) and lower median scores, suggesting stronger and more consistent predicted interactions. In contrast, miRNAs such as hsa-miR-4716 and hsa-miR-663b exhibited broader score distributions, indicating a wider range of target interaction strengths.

The corresponding high-confidence target genes and their context++ scores are detailed in Table 5. This table highlights the most likely biological targets of each miRNA and serves as the foundation for downstream functional enrichment analysis.

This graphical and tabular analysis offers an initial, non-parametric comparative overview of miRNA-specific target repression profiles, providing insights into their potential regulatory strength in the studied context.

### 2.6. Gene Ontology (GO) and Pathway Enrichment Results

**Figure 5:**
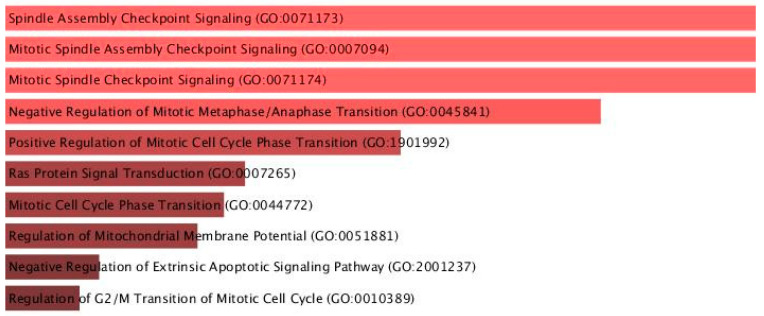
Top enriched GO biological processes of predicted miRNA targets visualized by bar chart.

To investigate the functional roles of the predicted miRNA target genes, Gene Ontology (GO) enrichment analysis was conducted using the Enrichr platform, focusing on the GO Biological Process 2021 database. The analysis revealed a significant overrepresentation of terms associated with cell cycle regulation, mitotic control, and apoptotic signaling pathways (Figure 5; Table 6).

Among the top-enriched biological processes were Spindle Assembly Checkpoint Signaling (GO:0071173), Mitotic Spindle Assembly Checkpoint Signaling (GO:0007094), and Mitotic Spindle Checkpoint Signaling (GO:0071174), all sharing an adjusted *p*-value of 0.00525 and a combined score of 364.80, along with Negative Regulation of Mitotic Metaphase/Anaphase Transition (GO:0045841; adjusted *p* = 0.00528, combined score = 326.76).

Other notable processes included Positive Regulation of Mitotic Cell Cycle Phase Transition, Ras Protein Signal Transduction, and Regulation of Mitochondrial Membrane Potential, all of which are mechanistically relevant to cellular stress responses, proliferation control, and programmed cell death.

The presence of high combined scores and low adjusted *p*-values supports the biological relevance of these enriched pathways. These results suggest that the selected miRNAs may exert their regulatory effects primarily through the modulation of the mitotic machinery and apoptotic regulation, highlighting their potential roles in cell cycle dysregulation associated with the studied condition.

### 2.7. Druggability Analysis of miRNA Target Genes

The full list of predicted high-confidence targets for hsa-miR-486-2, hsa-miR-664b-3p, and hsa-miR-6723-5p is provided in Supplementary Table S2. We conducted an in silico druggability analysis by mapping high-confidence miRNA targets—predicted via miRTarBase (v9.0) and TargetScanHuman (v8.0)—to DrugBank (v5.1.10) and DGIdb (https://dgidb.org/, accessed 21 June 2025).

hsa-miR-486-5p has been experimentally validated to target *PTEN* and *FOXO1* in human cell lines, both critical upstream regulators of the PI3K/Akt/mTOR pathway [29]; [miRTarBase]. This pathway is central to oxidative stress resilience in cochlear hair cells, and its modulation using Food and Drug Administration (FDA)-approved mTOR inhibitors—everolimus and temsirolimus—has demonstrated protective effects in inner ear models [30]. hsa-miR-664b-3p was linked to *TOP2A*, an essential gene for DNA replication and cell cycle progression. Inhibitors like etoposide induce DNA double-strand breaks and apoptosis through TOP2A poisoning, highlighting potential mechanisms to modulate cell survival in cochlear stress contexts [31]. hsa-miR-6723-5p is predicted via in silico databases [32,33] to target *SPARC, E2F1,* and *STAT3*, but experimental validation is currently lacking, particularly in a cochlear hair cell context, indicating a direction for future mechanistic studies.

Together, these confirmed and predicted miRNA–target–drug associations indicate multiple potential pharmacological entry points for modulating oxidative stress, apoptosis, and synaptic integrity in NIHL, thereby offering promising avenues for therapeutic intervention.

## 3. Discussion

### 3.1. Overview of Key Findings and Relevance to NIHL

In this study, we conducted a comprehensive miRNA expression profiling of individuals diagnosed with bilateral SNHL due to occupational noise exposure. A total of 72 transcripts were found to be differentially expressed, with 19 downregulated and 53 upregulated miRNAs and small RNAs. Among these, the most prominent candidates included upregulated miRNAs such as hsa-miR-4485, hsa-miR-501, hsa-miR-664b-3p, hsa-miR-486-2, and hsa-miR-663b, as well as downregulated ones including hsa-miR-6723, hsa-miR-194-2, hsa-miR-668-5p, hsa-miR-4722-3p, and hsa-miR-4716. These miRNAs were not only statistically significant in terms of fold change and FDR, but also demonstrated strong predicted interactions with gene targets implicated in cochlear stress responses, apoptotic regulation, and cell cycle progression.

### 3.2. Differential Expression and Molecular Impact of Key miRNAs

To uncover the molecular underpinnings of NIHL, we examined differentially expressed miRNAs in affected individuals. Several miRNAs exhibited significant dysregulation, suggesting their involvement in biological pathways associated with cochlear injury, including oxidative stress response, apoptosis regulation, synaptic remodeling, and immune signaling. These circulating miRNAs may reflect early molecular changes in the auditory system, functioning either as biomarkers of injury or as active mediators in cochlear pathophysiology.

NIHL primarily involves mechanical and metabolic stress on cochlear hair cells, which leads to excessive ROS production, the disruption of calcium homeostasis, and excitotoxic damage. These processes converge on apoptotic pathways and contribute to synaptic dysfunction and eventual hair cell loss [34,35]. Current studies have shown that the miR-183/96/182 cluster (the miR-183 family) is critically involved in the development of inner and outer hair cells, the formation of stereociliary bundles, and the maintenance of cellular homeostasis; dysregulation in the expression of this miRNA cluster has been found to directly impair hair cell function [20].

It is well established that intense noise exposure induces the excessive production of ROS, mitochondrial dysfunction, and the disruption of intracellular Ca^2+^ homeostasis in cochlear hair cells. These cellular stresses converge on apoptotic pathways involving kinases such as JNK and executioner caspases, ultimately leading to hair cell loss, synaptopathy, and the progressive degeneration of outer hair cells [10,11,35]. The role of miRNAs in this molecular cascade is supported by studies highlighting hair cell-specific miRNAs like miR-96, where point mutations in the miR-96 seed region have been directly linked to progressive hearing loss and impaired hair cell function in humans [17].

For example, a decrease in the expression of miR-183/96/182 cluster members has been observed following noise exposure; this reduction is correlated with disrupted stereocilia structure, increased apoptosis, and hair cell loss [21]. Moreover, evidence suggests that PTEN–PI3K/Akt signaling is critical for hair cell resilience, and the dysregulation of this pathway is associated with hair cell death; this signaling pathway may also be regulated by miRNAs [36].

Recent single-cell transcriptomic analyses have identified multiple transcriptional regulators that guide the diversification and resilience of spiral ganglion neurons. In particular, *NEUROD1* was found to act as a key determinant for the specification of Ic-type SGNs, highlighting the importance of tightly regulated gene networks in cochlear function [37]. In particular, transcriptional regulators such as *GFI1* not only maintain hair cell structural and functional identity under normal conditions but also repress neuronal gene programs that could otherwise trigger pathways leading to hair cell degeneration and apoptosis [38]. This regulatory capacity suggests that transcriptional regulators and their associated gene networks serve as integrators of developmental and environmental cues within the cochlear microenvironment, ensuring proper neuronal specification and resilience [37]. Future research employing single-cell transcriptomics or cell-type-specific expression profiling (e.g., RiboTag or spatial transcriptomics) holds promise for delineating the precise spatiotemporal dynamics of transcriptional responses in distinct cochlear cell populations and uncovering new strategies for enhancing hair cell resilience [39].

In addition to their diagnostic potential, the differentially expressed miRNAs identified in this study may offer actionable therapeutic insights through drug–gene interaction analysis. Among them, hsa-miR-486-2, predicted to target *PTEN* and *FOXO1*, intersects with the PI3K/Akt/mTOR axis—a pathway known to mediate oxidative stress resilience and hair cell survival in the cochlea. Recent findings highlight that mTORC2 activity is essential for stereocilia integrity and hair cell synaptic architecture, and its disruption leads to hair cell disorganization and functional decline [40,41]. The pharmacological modulation of the mTOR pathway using rapamycin has been shown to promote hair cell differentiation and delay age-related hearing loss, suggesting possible repurposing routes [42,43].

Similarly, hsa-miR-664b-3p is linked to *TOP2A*, a key regulator of DNA replication and mitotic progression. Etoposide, a classical TOP2A inhibitor, modulates DNA damage response pathways that are relevant for cell cycle regulation under stress conditions [44]. The dysregulation of synaptic integrity is a hallmark of noise-induced synaptopathy, and memantine, an NMDA receptor antagonist, has been shown to preserve inner hair cell synaptic ribbons and afferent connectivity in noise-exposed cochleae [40,45].

These mechanistically relevant interactions provide not only molecular validation of the observed miRNA shifts but also identify druggable nodes that could be leveraged for precision therapy in NIHL. These findings are supported by a detailed list of high-confidence targets shown in Supplementary Table S2, highlighting their regulatory influence on genes involved in oxidative stress, apoptosis, and synaptic maintenance. Integrating pharmacogenomic knowledge with transcriptomic insights may enhance future therapeutic strategies, particularly via local delivery approaches aimed at minimizing systemic side.

In our study, hsa-miR-194-2 was found to be significantly downregulated in individuals with NIHL. Although this miRNA has not yet been directly associated with NIHL in humans, Cao et al. demonstrated that miR-194 plays a crucial role in the development and differentiation of sensory patches and the statoacoustic ganglion in the zebrafish inner ear. Specifically, the loss of miR-194 function in zebrafish resulted in delayed inner ear development, abnormal otolith formation, disorganized sensory patch structure, and impaired neuronal patterning, ultimately leading to balance deficits and neurosensory dysfunction [46]. Taken together, these findings suggest that miR-194 may help maintain the anatomical and functional integrity of the auditory system during early development, which could be relevant to its downregulation in NIHL.

Complementarily, Du et al. reported that miR-194 modulates the differentiation of spiral ganglion neurons (SGNs) in neonatal mouse cochlear explants by targeting *RhoB*, a key regulator of cytoskeletal remodeling. They found that the overexpression of miR-194 enhanced SGN differentiation by increasing the proportion of TuJ1-positive cells and reducing Sox2-positive neural progenitors, suggesting the promotion of neuronal maturation [47].

PCA clearly separated patient and control samples in the scatter plot, suggesting robust transcriptional distinctions between the two groups. To further address the dimensional contribution of principal components in this study, we examined PC3, which accounted for 2.5% of the total variance. Although this percentage is modest compared to PC1 (80.1%) and PC2 (2.8%), PC3 captured unique variance that may reflect subtle yet biologically relevant differences within the patient group. Notably, Patient_12, which clustered closer to the control group in PC1–PC2 space, exhibited distinct loading along PC3, suggesting a divergent miRNA expression profile that may represent an intermediate or recovering molecular phenotype. The examination of PCA loading scores indicated that miRNAs such as hsa-miR-664b-3p and hsa-miR-4722-3p contributed disproportionately to PC3. Although these miRNAs have not yet been directly linked to auditory function, their predicted roles in mitotic control and synaptic regulation, as suggested by target prediction, align with biological processes that may influence cochlear repair and plasticity [48,49]. In line with the established findings that miRNAs modulate stress responses and cell-type-specific gene expression programs in cochlear sensory cells, these signals may represent individual-specific compensatory mechanisms in NIHL [50]. Therefore, PC3 may offer biological insights into sub-phenotypic variations within NIHL subjects, and its inclusion strengthens the interpretation of molecular heterogeneity observed in the PCA plot.

Taken together, our findings suggest that the downregulation of hsa-miR-194-2 may contribute to the increased vulnerability of the cochlear neurons by impairing developmental and synaptic stabilization pathways. This alteration might diminish neuronal resilience to noise-induced stress and disrupt homeostatic mechanisms critical for auditory processing. In addition to hsa-miR-194-2, several other differentially expressed miRNAs were identified as potential biomarker candidates, including hsa-miR-6723, hsa-miR-668-5p, hsa-miR-4722-3p, hsa-miR-4716, hsa-miR-4485, hsa-miR-501, hsa-miR-664b-3p, hsa-miR-486-2, and hsa-miR-663b. These miRNAs were selected based on their significant fold changes, low false discovery rates, and high-confidence target gene interactions. The functional enrichment analysis of their predicted target genes revealed significant involvement in biological processes such as the regulation of mitochondrial membrane potential, apoptotic signaling, oxidative stress response, cell cycle checkpoints, and MAPK signaling pathways—all of which are critically associated with cochlear stress responses and the pathophysiology of noise-induced hearing loss.

Among the miRNAs identified in our study, hsa-miR-6723-5p was found to be significantly downregulated in NIHL patients. Although direct auditory-related evidence for this miRNA remains limited, it has been implicated in modulating progenitor cell status and cellular stress responses in other systems. Its decreased expression may reflect a diminished regulatory capacity to support cochlear cell regeneration and resilience under noise-induced stress, potentially contributing to inner ear vulnerability [51].

Similarly, hsa-miR-668-5p exhibited significant downregulation in NIHL cases. Although direct evidence linking this miRNA to auditory function is currently lacking, it has been associated with mitochondrial dysfunction and oxidative stress regulation in various disease models, including neurodegenerative conditions such as Alzheimer’s disease (AD) [52]. Its downregulation in our NIHL cohort may reflect impaired redox homeostasis and mitochondrial defense—processes that are known to be critical for cochlear cell survival during acoustic trauma.

hsa-miR-4722-3p, which was significantly downregulated in NIHL patients in our study, has not yet been directly linked to auditory dysfunction. However, Liu et al. identified the dysregulation of its isoform, miR-4722-5p, in the peripheral blood of AD patients. The altered expression was associated with synaptic impairment and neuronal degeneration, implying a role in neurodegenerative processes. The observed downregulation of hsa-miR-4722-3p in our NIHL cohort may similarly reflect compromised synaptic maintenance or neuroprotective capacity in the cochlea, contributing to increased vulnerability to noise-induced injury [53]. Given the functional parallels between synaptic regulation in the brain and cochlea, its reduced expression in NIHL may signify a broader impairment in synaptic resilience mechanisms under noise-induced stress.

In our analysis, the predicted targets of hsa-miR-4722-3p included *ITPKB*, *TRAF1*, and *SYT12*, which participate in calcium signaling, TNF-mediated neuroinflammation, and synaptic vesicle trafficking—processes known to influence cochlear vulnerability. These functional associations raise the possibility that hsa-miR-4722-3p may contribute to the stress-related modulation of auditory signaling pathways following noise exposure. Likewise, hsa-miR-4716 has emerged as another candidate of interest. In one study, the GT genotype of the rs2304186 SNP located in the 3′-UTR region of the *AKT2* gene was shown to alter the binding affinity of hsa-miR-4716-3p, potentially increasing susceptibility to NIHL. This SNP is thought to disrupt the interaction between has-miR-4716-3p and AKT2 mRNA, resulting in *AKT2* overexpression and impairments in cellular stress response pathways [54].

Recent studies have highlighted hsa-miR-4485-3p as a regulator of cellular fate in stress contexts. Cheng et al. showed that miR-4485-3p, enriched in apoptotic vesicles from mesenchymal stem cells, suppresses osteogenesis and enhances adipogenesis by modulating AKT pathway signaling. This shift is tightly linked to mitochondrial dynamics, redox balance, and apoptosis regulation. Although this was conducted in non-auditory tissues, these pathways—especially AKT-mediated cell survival, mitochondrial integrity, and oxidative stress response—are critically implicated in cochlear hair cell maintenance and injury recovery [55]. In our study, hsa-miR-4485 was upregulated in NIHL patients, suggesting it may play a role in orchestrating stress-response programs in cochlear cells. Elevated levels could reflect the compensatory activation of AKT-mediated survival mechanisms or, conversely, contribute to maladaptive regulation that impairs hair cell regeneration. While the exact function of miR-4485 in the cochlea remains to be established, its known roles in apoptosis and mitochondrial signaling make it a compelling candidate for further investigation in the context of noise-induced auditory damage.

The association of hsa-miR-501-3p with synaptic plasticity and neurodegeneration supports its potential involvement in conditions such as NIHL. miR-501-3p has been shown to directly target the 3′-UTR of *Gria1*, encoding the GluA1 AMPA receptor subunit, resulting in reduced GluA1 protein expression. Notably, its expression is upregulated locally in dendrites following NMDA receptor stimulation, and this increase is required for the NMDA-induced downregulation of GluA1 and long-lasting dendritic spine remodeling [56]. These mechanisms—particularly synaptic strength regulation and dendritic stability—are critical in the cochlea for maintaining afferent synapse integrity. Accordingly, the upregulation of hsa-miR-501-3p observed in our NIHL cohort may reflect an adaptive or dysregulated synaptic response to noise-induced stress.

In our dataset, hsa-miR-664b-3p was identified as significantly upregulated in NIHL patients and a top contributor to PC2-driven separation between the patient and control groups. Although a direct link to auditory pathology is yet to be established, preclinical studies highlight its broader relevance in cellular stress responses. For instance, miR-664b-3p has been shown to suppress tumor cell proliferation and migration by targeting BUB3, a mitotic checkpoint protein [57]. Additionally, miR-664b-3p expression correlates with extended cellular lifespan and modulation of MAPK and FoxO pathways—the key regulators of oxidative stress and apoptosis [58].

Hsa-miR-664b-3p has been implicated in cell cycle control and apoptosis regulation. Zhao et al. demonstrated that miR-664b-3p inhibits colon carcinoma cell proliferation and promotes apoptosis by targeting *Bub3*, a mitotic checkpoint protein involved in cell cycle progression [57]. Although its role in auditory systems remains uncharacterized, these findings suggest miR-664b-3p may influence cellular stress responses and warrants further investigation in other pathophysiological contexts, including NIHL.

In our study, hsa-miR-663b was found to be significantly upregulated in NIHL patients. Although no direct link between hsa-miR-663b and NIHL has been established in the current literature, several studies have highlighted its role in biological processes that may be relevant to cochlear stress response and injury. For instance, in colorectal cancer, hsa-miR-663b promotes tumor cell proliferation and migration by targeting *APC2* and activating the Wnt/β-catenin signaling pathway [59]. In cervical cancer, the exosomal transfer of hsa-miR-663b into endothelial cells was shown to suppress vinculin, thereby facilitating angiogenesis [60]. Both Wnt/β-catenin signaling and cytoskeletal regulation are implicated in cell survival, synaptic integrity, and reparative mechanisms in hair cells. Based on these functional insights, the upregulation of miR-663b in our NIHL subjects may reflect an attempt at cytoskeletal remodeling or angiogenic response within the cochlea following acoustic injury. This positions hsa-miR-663b as a promising candidate for further mechanistic studies into its role in cochlear homeostasis and NIHL pathogenesis [61].

Collectively, the convergence of these miRNAs on shared pathways such as oxidative stress regulation, apoptosis, mitochondrial signaling, and cytoskeletal remodeling underscores their biological relevance to NIHL and supports their potential utility as biomarkers and therapeutic targets.

### 3.3. Biological Pathways and GO Enrichment: Cell Cycle and Apoptosis

The GO enrichment analysis of the high-confidence predicted targets of differentially expressed miRNAs revealed strong associations with key biological processes implicated in cellular stress regulation and cochlear pathology. Among the most significantly enriched terms were Spindle Assembly Checkpoint Signaling (GO:0071173), Mitotic Spindle Assembly Checkpoint Signaling (GO:0007094), and Mitotic Spindle Checkpoint Signaling (GO:0071174), all of which shared an adjusted *p*-value of 0.00525 and a combined enrichment score of 364.80. These pathways are essential for maintaining genomic integrity during cell division and are tightly regulated under stress conditions to prevent mitotic errors that could lead to cell death or dysfunction. Another highly enriched process, Negative Regulation of Mitotic Metaphase/Anaphase Transition (GO:0045841; adjusted *p* = 0.00528), suggests the activation of checkpoint pathways that may halt the cell cycle in response to persistent cochlear damage. This aligns with findings in sensorineural hearing loss models, where DNA damage and mitotic disruption have been observed in supporting cells of the inner ear following noise exposure [12].

In parallel, Regulation of Mitochondrial Membrane Potential (GO:0051881) and Negative Regulation of Extrinsic Apoptotic Signaling Pathway (GO:2001237) were among the top enriched terms, pointing to a convergence on mitochondrial function and apoptosis. These processes are well-established contributors to outer hair cell vulnerability in NIHL and are central to the transition from reversible cellular stress to irreversible degeneration [62]. Given the sensitivity of cochlear outer hair cells to oxidative and mitotic stress, the enrichment of these GO categories supports the hypothesis that dysregulated miRNAs in NIHL may serve as the key modulators of apoptosis, cell cycle arrest, and mitochondrial dysfunction. These mechanisms are critical not only for understanding the molecular basis of noise-induced damage but also for identifying potential therapeutic intervention points [63].

### 3.4. Diagnostic and Prognostic Utility of C-miRNAs

C-miRNAs such as hsa-miR-486-2, hsa-miR-664b-3p, and hsa-miR-668-5p emerged in our study as promising minimally invasive biomarkers for the early detection and stratification of NIHL. Although these specific miRNAs have not been extensively studied in auditory tissues, their known roles in oxidative stress, mitochondrial function, and apoptotic regulation in other systems suggest plausible mechanistic relevance. Prior studies have shown that miRNA expression profiles in serum and perilymph can correlate with auditory thresholds and cochlear cell integrity [64], supporting their potential clinical utility.

In our study, unsupervised hierarchical clustering and PCA mapping revealed distinct segregation between the NIHL and control groups, indicating robust miRNA expression signatures associated with cochlear pathology. This molecular stratification highlights the potential of miRNA-based approaches for patient classification and individualized risk assessment. Notably, Patient_12, whose expression profile clustered more closely with controls, may represent a case of subclinical or early-stage NIHL or a phenotype with enhanced endogenous protective mechanisms—underscoring the relevance of miRNA profiling for uncovering intra-group variability and prognostic differentiation.

Furthermore, the identified miRNAs are functionally linked to oxidative stress regulation, apoptosis, synaptic remodeling, and mitochondrial homeostasis—all of which are mechanistically relevant to the progression of NIHL. This functional convergence supports the clinical utility of these circulating miRNAs as dynamic molecular indicators of disease presence, severity, and potentially therapeutic response.

### 3.5. Functional Network Complexity Beyond miRNAs

In addition to miRNAs, our analysis identified the significant dysregulation of other small non-coding RNAs, including snoRNAs and scaRNAs, highlighting a broader post-transcriptional regulatory complexity in NIHL beyond canonical miRNA–mRNA interactions.

Among the most prominently upregulated snoRNAs were U96a and U96b, both belonging to the H/ACA box class. These snoRNAs guide the pseudouridylation of 18S and 28S rRNA and are essential for proper ribosome biogenesis and function. Their overexpression may reflect a compensatory mechanism to maintain translational fidelity during cellular stress in the cochlea [65]. Another H/ACA snoRNA, ACA32, showed a 4.29-fold increase in NIHL subjects. ACA32 plays a role in rRNA pseudouridylation and ribonucleoprotein complex assembly, and its dysregulation may influence ribosome remodeling and stress-related translational control [66]

Conversely, ACA50 was significantly downregulated in our NIHL cohort (−2.27-fold change), suggesting the possible impairment of rRNA modification pathways. The disruption of ACA50 expression has been linked to abnormal pre-rRNA processing and cellular vulnerability to oxidative and metabolic stress [67]. Additionally, *SNORA38B* (upregulated by 3.79-fold) has been implicated in regulating apoptosis and cell cycle checkpoints through its involvement in rRNA pseudouridylation machinery, particularly in stress conditions and malignant transformation [68].

Another notable transcript, HBII-166, a box C/D snoRNA, demonstrated more than fivefold upregulation. Although its function is less well characterized, similar snoRNAs have been shown to participate in the 2′-O-methylation of rRNA and contribute to translational control in stress contexts [69]. Further, ACA3-2, ACA41, and U3 snoRNAs were all significantly elevated in NIHL patients. U3, a well-studied box C/D snoRNA, initiates early pre-rRNA cleavage during ribosome assembly. Its overexpression may represent an attempt to restore or adapt ribosome production under cellular injury [70,71].

Together, these findings demonstrate that snoRNAs involved in ribosomal RNA modification and biogenesis are differentially expressed in NIHL. Their dysregulation may influence the key aspects of translational control, mitochondrial metabolism, and cellular resilience in the cochlea. This highlights an underexplored layer of regulatory complexity in NIHL pathogenesis and suggests that specific snoRNAs could serve as novel biomarkers or therapeutic targets in auditory injury.

### 3.6. Study Limitations and Future Directions

While this study has certain limitations typical of exploratory biomarker research, the multi-step bioinformatic validation and integration with known biological pathways provide a solid foundation for future translational work. The relatively small sample size may reduce statistical power and limit generalizability. Although all samples met QC standards at the probe level, subtle transcriptional differences related to patient comorbidities may persist and could influence expression variance. This was not the primary focus of the current analysis but remains an important consideration for future stratified transcriptomic studies. A limitation of this study is that stratified QC analyses by comorbidity status were not performed due to the sample size; however, all comorbid cases passed individual probe-level QC thresholds, minimizing potential bias. Additionally, the cohort’s demographic homogeneity—comprising only male participants from a specific occupational background—may not fully represent the broader NIHL population.

Although microarray-based profiling enabled the robust identification of differentially expressed miRNAs and small RNAs, experimental validation remains essential. Future studies should employ luciferase reporter assays, miRNA overexpression or inhibition experiments, and possibly CRISPR-based methods to verify predicted miRNA–target interactions and their downstream functional effects.

Another important consideration is the lack of longitudinal data. Capturing dynamic changes in miRNA expression following acoustic trauma across multiple time points would better reflect the temporal evolution of cochlear injury and recovery. Furthermore, integrating additional omics layers—such as epigenomics, proteomics, and metabolomics—would deepen our understanding of how molecular networks converge to shape NIHL pathogenesis. Lastly, the exploration of extracellular vesicle-associated miRNAs, particularly in the serum or perilymph, may offer further insights into intercellular signaling mechanisms and provide highly stable, non-invasive diagnostic candidates.

Moreover, patient-level comorbidities and possible unilateral involvement, such as observed in Patient_12, may introduce subtle variability that warrants stratified analyses in future studies

## 4. Materials and Methods

### 4.1. Clinical Specimens

Twelve male patients diagnosed with occupational noise-induced hearing loss were recruited from the Istanbul Yedikule Chest Diseases and Chest Surgery Training and Research Hospital and the Acıbadem Kartal Hospital Ear, Nose, and Throat Polyclinic. All patients were employed in high-exposure industries, such as automotive and textile manufacturing. Six age-matched healthy male volunteers with no clinical signs of hearing impairment served as the control group. The age range for patients with hearing loss was 39 to 54 years, while that of the control participants was 35 to 52 years.

All study participants were male. Ethical approval for this study was obtained from the Clinical Research Ethics Committee of Istanbul Medical Faculty on 24 January 2023 (Approval No: E-29624016-050.99-1591715). Written informed consent was obtained from all individuals prior to enrollment.

Based on a sample size calculation considering a log2 fold change (FC) of 2.00–2.2 and a standard deviation (SD) of approximately 1.0 for any miRNA between the patient and control groups, with a power of 0.80 and a significance level (α) of 0.05, the minimum required sample size was estimated to be 6–7 individuals per group. Accordingly, 12 individuals were included in the study group and 6 in the control group.

### 4.2. Sample Processing and Quality Control

Total RNA samples were processed using the Affymetrix GeneChip Human Transcriptome Array 2.0 (HTA 2.0) platform (Affymetrix, Thermo Fisher Scientific, Santa Clara, CA, USA), following the FlashTag Biotin HSR labeling protocol (Thermo Fisher Scientific (Frederick, MD, USA), MAN0018109). All RNA samples were evaluated for purity and integrity using NanoDrop (Thermo Fisher Scientific, Wilmington, DE, USA) and Agilent Bioanalyzer 2100 platforms (Agilent Technologies, Santa Clara, CA, USA). Only samples with RIN ≥7 were included in the study.

Comprehensive quality control (QC) measures were implemented at both the probe and array levels. QC was performed using the Affymetrix Expression Console and Transcriptome Analysis Console (TAC) software. Metrics such as average background, signal distribution, percentage of present calls, hybridization controls, and RNA degradation profiles were systematically assessed. Arrays that did not meet the platform’s QC thresholds were excluded from downstream processing. These procedures ensured accurate biotin labeling, target fragmentation, and hybridization quality.

Although comorbidity status was documented in clinical metadata, transcriptome QC metrics were not stratified based on these conditions due to sample size limitations. Importantly, all included samples passed stringent QC criteria, minimizing potential variability introduced by underlying clinical heterogeneity [Thermo Fisher Scientific. FlashTag™ Biotin HSR RNA Labeling Kit User Guide. MAN0018109. Available at: https://assets.thermofisher.com/TFS-Assets%2FLSG%2Fmanuals%2FMAN0018109-FlashtagBiotinHSR-GeneChipArrays-UG.pdf (accessed on 22 June 2025)].

Additionally, individual probe-level QC was performed for all patients using the Affymetrix Expression Console (v1.4.1.46; Affymetrix, Thermo Fisher Scientific, Santa Clara, CA, USA) and TAC software (v4.0.3.14; Affymetrix, Thermo Fisher Scientific, Santa Clara, CA, USA). Metrics such as pm mean, average background, and hybridization control signals were assessed. Patients with comorbidities (IDs 4, 6, 8, 9, 11) passed the same strict QC thresholds as other subjects without any outlier signals. The detailed values are provided in Supplementary Table S1.

### 4.3. Total RNA Isolation from Blood Samples

Total RNA was extracted from whole blood samples in accordance with the manufacturer’s instructions using the QiaAmp RNA Blood Mini Kit (Cat. No. 52304; Qiagen, Hilden, Germany). RNA concentration and purity were assessed with a NanoDrop 2000c spectrophotometer (Thermo Fisher Scientific, Wilmington, DE, USA), with acceptable A260/A280 ratios ranging from 1.8 to 2.0.

### 4.4. MicroRNA Expression Array

The GeneChip^®^ miRNA 4.0 Array (Affymetrix) includes the comprehensive coverage of human small RNAs based on miRBase v20. For *Homo sapiens*, the array comprises probe sets for 2578 mature human miRNAs, along with a curated set of small nucleolar RNAs (snoRNAs), small Cajal body-specific RNAs (scaRNAs), and small nuclear RNAs (snRNAs). These additional RNA classes are included to enhance the functional annotation of non-coding RNA profiles and support broader regulatory network analyses. The inclusion of both predicted and experimentally validated targets enables the high-confidence downstream interpretation of miRNA-related regulatory mechanisms.

Microarray analysis was performed using the GeneChip^®^ miRNA 4.0 Array (Affymetrix, Santa Clara, CA, USA; Cat. No. 902411) following the manufacturer’s protocols. This array platform enables the comprehensive profiling of known miRNAs from multiple species, including human, based on the miRBase database. Briefly, 130 ng of total RNA, including small RNAs, was first labeled using the FlashTag™ Biotin HSR RNA Labeling Kit (Thermo Fisher Scientific, Frederick, MD, USA; Pub. No. MAN0018109). The labeled RNA was then hybridized to the GeneChip^®^ miRNA 4.0 Array in a hybridization oven at 48 °C for 16 h.

Following hybridization, arrays were washed and stained using the Affymetrix GeneChip^®^ Fluidics Station 450 (Affymetrix, Thermo Fisher Scientific, Santa Clara, CA, USA) and scanned with the GeneChip^®^ Scanner 3000 7G (Affymetrix, Thermo Fisher Scientific, Santa Clara, CA, USA). Raw intensity data (CEL files) were extracted using the Affymetrix GeneChip^®^ Command Console Software (v4.0.0.1567; Affymetrix, Thermo Fisher Scientific, Santa Clara, CA, USA). Quality control, normalization (using Robust Multi-array Average—RMA), and downstream statistical analyses were conducted using the TAC Software or appropriate R/Bioconductor packages. miRNAs showing statistically significant differential expression between the groups were selected based on adjusted *p*-values (e.g., FDR < 0.05) and fold change thresholds. 

### 4.5. Statistical Analysis

In the presentation of the data, descriptive statistics were used by giving categorical data as numbers and percentages and continuous data as mean and standard deviation values. In the comparison of mean values between two groups in continuous variables with normal distribution, the *t*-test was used in independent groups, and the Mann–Whitney U test was used in groups without normal distribution. Data were analyzed with the support of the SPSS 28 statistical package program; statistical significance was accepted as *p* < 0.05 and two-sided.

### 4.6. miRNA–Target Prediction and Score Distribution Analysis

The prediction of miRNA–target interactions was performed using the TargetScan 8.0 database, which provides cumulative weighted context++ scores for each predicted target gene. These scores reflect the predicted binding affinity between a miRNA and its mRNA target; lower (more negative) values indicate stronger predicted repression.

For each selected miRNA, the top 10 predicted target genes with the lowest cumulative context++ scores were extracted from TargetScan output files. These genes were then compiled into a single dataset to enable comparative analysis across all miRNAs included in the study.

To visualize the distribution and variability of predicted binding strengths, a boxplot was generated using the Python data visualization library Seaborn (v0.11.2), along with Matplotlib (v3.7.1) and Pandas (v1.5.3) in a Python 3.10.12 environment. The boxplot illustrated the spread of context++ scores for each miRNA, highlighting differences in median values, interquartile ranges (IQRs), and potential outliers. No formal statistical test was applied in this step, as the purpose of the visualization was exploratory—to assess the relative strength and consistency of predicted interactions among different miRNAs.

### 4.7. Gene Ontology and Functional Enrichment Analysis

To explore the biological functions associated with the predicted target genes of the selected miRNAs, functional enrichment analysis was performed using the Enrichr platform (https://maayanlab.cloud/Enrichr/ (accessed on 10 May 2025)). Gene lists were prepared by compiling the top predicted targets (based on the lowest cumulative context++ scores) for each miRNA, resulting in a combined high-confidence target gene set.

This gene set was submitted to Enrichr, and the “GO Biological Process 2021” library was selected for analysis. Additional enrichment analyses were also conducted using KEGG 2021 Human and Reactome 2022 pathway databases to identify significantly over-represented pathways. The statistical significance of each enriched term was calculated by Enrichr using a combination of Fisher’s exact test and adjusted *p*-values based on the Benjamini–Hochberg FDR correction. GO terms and pathways with adjusted *p*-values (FDR) < 0.05 were considered statistically significant.

The visualization of enrichment results was carried out using the bar chart output provided by Enrichr, which ranks GO terms based on the combined score (a metric that accounts for both significance and magnitude of enrichment). The top 10 enriched GO terms were highlighted and used for further biological interpretation.

### 4.8. In Silico miRNA–mRNA Interaction and Drug Repositioning Analysis

To explore the biological implications of differentially expressed miRNAs, high-confidence target genes were predicted using the established interaction databases including miRTarBase (v9.0) (https://miRTarBase.cuhk.edu.cn/, accessed on 22 June 2025) and TargetScanHuman (v8.0) (https://www.targetscan.org/vert_80/, accessed on 22 June 2025). Only conserved targets with experimental validation or high context++ scores were included in downstream analyses.

To investigate the potential for therapeutic targeting, identified target genes were queried against DrugBank (v5.1.10) (https://go.drugbank.com/, accessed on 22 June 2025) and the Drug–Gene Interaction Database (DGIdb) (https://www.dgidb.org/, accessed on 22 June 2025) to retrieve known and investigational compounds modulating these targets. Only FDA-approved or clinically relevant drug–gene interactions were reported.

This approach enabled the identification of druggable miRNA–mRNA axes with potential relevance to NIHL pathogenesis and therapy.

## 5. Conclusions

In summary, this study provides novel insights into the molecular landscape of NIHL by identifying a distinct panel of differentially expressed circulating miRNAs. Notably, miRNAs such as hsa-miR-486-2, hsa-miR-664b-3p, and hsa-miR-6723-5p emerged as promising candidates with potential diagnostic and therapeutic relevance. These miRNAs were associated with key biological processes implicated in cochlear stress and injury, including apoptotic signaling, cell cycle regulation, oxidative stress response, and mitochondrial dysfunction.

The functional enrichment analysis of their predicted targets supported the hypothesis that these miRNAs play pivotal roles in modulating cellular vulnerability, survival pathways, and synaptic integrity under acoustic trauma. The integration of hierarchical clustering, PCA mapping, and GO pathway analysis further strengthens the clinical potential of these biomarkers in detecting early molecular alterations in NIHL.

Overall, our findings support the application of circulating miRNAs as minimally invasive molecular indicators of cochlear injury. Further mechanistic and longitudinal studies are warranted to validate their utility and explore their application in precision medicine approaches aimed at preventing or mitigating noise-induced auditory damage.

## Figures and Tables

**Table 1:** Hearing thresholds at different frequencies in NIHL and control groups.

Hearing Loss Group	0.5 kHz	1 kHz	2 kHz	4 kHz	6 kHz	8 kHz
Ear	M (SD)	M (SD)	M (SD)	M (SD)	M (SD)	M (SD)
Right Ear	28.75	29.58 *	39.17 *	57.08 ¶	52.50 ¶	58.33 ¶
	17.07	17.90	21.30	17.25	21.69	21.99
Left Ear	33.75 *	33.33	44.17 *	53.92 ¶	53.75 ¶	56.67 ¶
	30.83	31.65	30.88	19.27	24.23	27.74
**Control Group**	16.67	13.33	14.17	12.50	13.33	11.67
Right Ear	2.58	2.58	3.76	2.74	2.58	2.58
Left Ear	13.33	11.67	13.33	11.67	12.50	11.67
	2.58	2.58	2.58	2.58	2.74	2.58

M: mean, SD: standard deviation; * *p* < 0.05; ¶ *p* < 0.001.

**Table 2:** Log2 expression, fold change, and FDR-adjusted *p*-values of differentially expressed miRNAs and small RNAs.

ID	Control Avg (log2)	Patient Avg (log2)	Fold Change	*p*-Val	FDR *p*-Val	Transcript ID (Array Design)
20537465	2.58	1.06	−2.86	1.84 × 10^−9^	1.22 × 10^−5^	hsa-mir-6723
20534809	2.23	1.09	−2.21	1.55 × 10^−8^	5.13 × 10^−5^	hsa-mir-194-2
20536918	2.48	1.44	−2.05	1.49 × 10^−6^	0.001	hsa-mir-1343
20538277	2.14	3.23	2.13	5.80 × 10^−6^	0.002	U96b
20504554	2.49	1.34	−2.22	6.79 × 10^−6^	0.002	hsa-miR-668-5p
20538276	4.94	7.05	4.32	6.98 × 10^−6^	0.002	U96a
20533389	2.89	1.65	−2.35	1.27 × 10^−5^	0.0028	ENSG00000238581
20536757	2.39	4.57	4.54	1.47 × 10^−5^	0.0031	hsa-mir-4485
20535205	2.83	4.09	2.39	2.80 × 10^−5^	0.0046	hsa-mir-501
20532687	2.29	1.11	−2.27	4.40 × 10^−5^	0.0063	ACA50
20538104	2.32	4.24	3.79	4.49 × 10^−5^	0.0063	*SNORA38B*
20537539	2.49	1.39	−2.14	6.60 × 10^−5^	0.0081	hsa-mir-6796
20519556	2.01	0.88	−2.19	0.0002	0.0162	hsa-miR-4722-3p
20534225	2.44	4.79	5.1	0.0003	0.021	HBII-166
20521811	1.61	3.94	5.03	0.0005	0.026	hsa-miR-664b-3p
20534600	1.92	3.08	2.23	0.0005	0.0261	hsa-mir-142
20537879	4.02	5.15	2.19	0.0007	0.0305	hsa-mir-486-2
20532682	2.44	1.36	−2.12	0.0008	0.0318	ACA47
20532647	1.97	3.83	3.63	0.0008	0.032	ACA3-2
20536948	3.98	2.62	−2.58	0.0008	0.032	hsa-mir-4716
20533559	2.34	1.32	−2.03	0.0008	0.0327	ENSG00000238852
20532706	2.35	3.53	2.26	0.0009	0.0331	ACA62
20538129	2.79	1.63	−2.22	0.001	0.0352	U108
20538128	2.82	1.73	−2.12	0.001	0.0357	U108
20505964	1.94	0.93	−2.02	0.0013	0.042	hsa-miR-924
20538224	4.7	6.14	2.71	0.0018	0.0508	U68
20506797	1.34	2.45	2.16	0.0027	0.0627	hsa-miR-663b
20532669	2.96	4.29	2.51	0.0031	0.0667	ACA40
20534329	3.28	1.79	−2.8	0.0033	0.0706	HBII-85-6
20538223	6.33	7.4	2.11	0.0035	0.0727	U68
20532651	2.34	4.44	4.29	0.0039	0.0755	ACA32
20536823	3.29	2.22	−2.09	0.0045	0.0792	hsa-mir-4539
20532671	2.41	4.67	4.79	0.0045	0.0798	ACA41
20532621	2.06	3.07	2.01	0.0055	0.0905	ACA13
20538125	3.7	5.59	3.7	0.0063	0.0982	U105
20538208	2.74	1.71	−2.05	0.0069	0.1032	U58A
20538203	4.54	5.64	2.14	0.0069	0.1032	U54
20525457	3.09	1.8	−2.45	0.0078	0.1093	hsa-miR-6748-5p
20532648	2.8	3.9	2.14	0.0087	0.1142	ACA3-2
20538109	8.51	9.94	2.68	0.0109	0.1248	*SNORD119*
20532670	2.43	1.34	−2.12	0.0111	0.1255	ACA41
20500128	10.29	11.31	2.03	0.0121	0.1331	hsa-miR-16-5p
20538175	4.82	6.51	3.21	0.014	0.1434	U3
20534374	2.74	3.79	2.08	0.0152	0.1499	hsa-mir-28
20518444	2.87	4.27	2.64	0.0171	0.1567	hsa-miR-3150b-3p
20500151	8.97	10.03	2.08	0.0184	0.1635	hsa-miR-25-3p
20534249	4.45	5.47	2.03	0.0211	0.1751	HBII-436
20534244	2.93	4.24	2.47	0.0211	0.1751	HBII-336
20534235	4.68	6.36	3.2	0.0223	0.1801	HBII-251
20534242	2.69	4.42	3.3	0.0242	0.1885	HBII-296B
20532652	2.05	3.25	2.3	0.0245	0.1898	ACA32
20538325	4.72	6.48	3.39	0.0262	0.1968	U3-2B
20538326	4.72	6.48	3.39	0.0262	0.1968	U3-2
20538327	4.72	6.48	3.39	0.0262	0.1968	U3-3
20538328	4.72	6.48	3.39	0.0262	0.1968	U3-4
20538173	6.98	8.29	2.49	0.0266	0.1982	U38B
20538165	8.5	9.72	2.34	0.0267	0.1988	U34
20538171	6.98	8.14	2.23	0.0272	0.2009	U37
20500488	6.33	8.05	3.3	0.0284	0.2053	hsa-miR-223-3p
20534241	2.65	4.4	3.38	0.0299	0.2092	HBII-296B
20538199	9.44	10.95	2.85	0.0306	0.2102	U50
20538304	6.43	7.77	2.55	0.032	0.2157	snR39B
20525436	2.54	3.61	2.1	0.0327	0.2183	hsa-miR-6737-5p
20506004	3.52	5.06	2.89	0.0329	0.2186	hsa-miR-935
20504364	5.04	6.33	2.45	0.0333	0.2193	hsa-miR-619-5p
20500442	1.25	2.59	2.53	0.0374	0.2336	hsa-miR-34a-5p
20538209	8.57	9.83	2.39	0.038	0.2356	U58B
20500769	4.81	7.04	4.7	0.0392	0.2387	hsa-miR-126-3p
20532632	4.77	6.18	2.67	0.0426	0.2471	ACA20
20538282	6.49	7.61	2.16	0.0426	0.2471	Z17B
20518834	6.85	7.96	2.16	0.0435	0.2502	hsa-miR-4454
20504584	2.63	4.04	2.66	0.0469	0.2607	hsa-miR-378d

**Table 3:** Top miRNAs contributing to PC2 separation based on loading scores.

Rank	miRNA ID	PC2 Loading Score *
1	hsa-miR-486-2	0.1028
2	hsa-miR-4722-3p	−0.0983
3	hsa-miR-194-2	−0.0914
4	hsa-miR-664b-3p	0.0885
5	hsa-miR-501	0.0867
6	hsa-miR-663b	0.0791
7	hsa-miR-4716	−0.0756
8	hsa-miR-6723	−0.0719
9	hsa-miR-668-5p	−0.0684
10	hsa-miR-4485	0.0651

* Negative values reflect downregulated miRNAs in the NIHL group contributing to the PC2 axis, while positive values indicate upregulated miRNAs.

**Table 4:** Potential biomarker candidates.

miRNA ID	Fold Change	*p*-Value	FDR	Regulation
hsa-miR-6723	−2.86	1.84 × 10^−9^	1.22 × 10^−5^	↓ Down
hsa-miR-194-2	−2.21	1.55 × 10^−8^	5.13 × 10^−5^	↓ Down
hsa-miR-668-5p	−2.22	6.79 × 10^−6^	0.002	↓ Down
hsa-miR-4722-3p	−2.19	0.0002	0.0162	↓ Down
hsa-miR-4716	−2.58	0.0008	0.032	↓ Down
hsa-miR-4485	4.54	1.47 × 10^−5^	0.0031	↑ Up
hsa-miR-501	2.39	2.80 × 10^−5^	0.0046	↑ Up
hsa-miR-664b-3p	5.03	0.0005	0.026	↑ Up
hsa-miR-486-2	2.19	0.0007	0.0305	↑ Up
hsa-miR-663b	2.16	0.0027	0.0627	↑ Up

**Table 5:** List of top predicted miRNA targets with lowest cumulative context++ scores.

miRNA	Target Gene	Gene Name	Cumulative Weighted Context++ Score
hsa-miR-501-3p/502-3p	*RPRD1B*	regulation of nuclear pre-mRNA domain containing 1B	−0.78
hsa-miR-501-3p/502-3p	*MYNN*	myoneurin	−0.64
hsa-miR-501-3p/502-3p	*ADAMTS3*	ADAM metallopeptidase with thrombospondin type 1 motif, 3	−0.55
hsa-miR-501-3p/502-3p	*ARPP21*	cAMP-regulated phosphoprotein, 21kDa	−0.55
hsa-miR-501-3p/502-3p	*SAMD12*	sterile alpha motif domain containing 12	−0.54
hsa-miR-501-3p/502-3p	*B4GALT5*	UDP-Gal:betaGlcNAc beta 1,4-galactosyltransferase, polypeptide 5	−0.53
hsa-miR-501-3p/502-3p	*SEC63*	SEC63 homolog (*S. cerevisiae*)	−0.52
hsa-miR-501-3p/502-3p	*CLIC4*	chloride intracellular channel 4	−0.47
hsa-miR-501-3p/502-3p	*JDP2*	Jun dimerization protein 2	−0.47
hsa-miR-501-3p/502-3p	*COL10A1*	collagen, type X, alpha 1	−0.47
hsa-miR-501-5p	*XKR4*	XK, Kell blood group complex subunit-related family, member 4	−1.11
hsa-miR-501-5p	*KLHDC1*	kelch domain containing 1	−0.99
hsa-miR-501-5p	*PEX12*	peroxisomal biogenesis factor 12	−0.94
hsa-miR-501-5p	*NLRP11*	NLR family, pyrin domain containing 11	−0.87
hsa-miR-501-5p	*SPRR2A*	small proline-rich protein 2A	−0.83
hsa-miR-501-5p	*SPRR2F*	small proline-rich protein 2F	−0.81
hsa-miR-501-5p	*HTN3*	histatin 3	−0.78
hsa-miR-501-5p	*SPRR2E*	small proline-rich protein 2E	−0.76
hsa-miR-501-5p	*CLCA4*	chloride channel accessory 4	−0.74
hsa-miR-501-5p	*LPAR1*	lysophosphatidic acid receptor 1	−0.71
hsa-miR-663b	*BSPRY*	B-box and SPRY domain containing	−1.17
hsa-miR-663b	*GPRIN2*	G protein regulated inducer of neurite outgrowth 2	−1.05
hsa-miR-663b	*CSF2*	colony-stimulating factor 2 (granulocyte–macrophage)	−1
hsa-miR-663b	*C1orf158*	chromosome 1 open reading frame 158	−0.9
hsa-miR-663b	*LL22NC03-75H12.2*	novel protein; uncharacterized protein	−0.81
hsa-miR-663b	*OSBPL5*	oxysterol binding protein-like 5	−0.81
hsa-miR-663b	*RIBC2*	RIB43A domain with coiled-coils 2	−0.79
hsa-miR-663b	*PYCR1*	pyrroline-5-carboxylate reductase 1	−0.77
hsa-miR-663b	*CDKN2A*	cyclin-dependent kinase inhibitor 2A	−0.71
hsa-miR-663b	*SHARPIN*	SHANK-associated RH domain interactor	−0.7
hsa-miR-668-5p	*IER3*	immediate early response 3	−1.43
hsa-miR-668-5p	*SEPT5*	septin 5	−0.86
hsa-miR-668-5p	*NNAT*	neuronatin	−0.84
hsa-miR-668-5p	*MED10*	mediator complex subunit 10	−0.84
hsa-miR-668-5p	*TSSK6*	testis-specific serine kinase 6	−0.78
hsa-miR-668-5p	*TXNL4B*	thioredoxin-like 4B	−0.73
hsa-miR-668-5p	*C1orf216*	chromosome 1 open reading frame 216	−0.72
hsa-miR-668-5p	*NSUN5*	NOP2/Sun domain family, member 5	−0.7
hsa-miR-668-5p	*LDOC1L*	leucine zipper, down-regulated in cancer 1-like	−0.69
hsa-miR-668-5p	*GBGT1*	globoside alpha-1,3-N-acetylgalactosaminyltransferase 1	−0.68
hsa-miR-4485-3p	*PARK7*	parkinson protein 7	−0.92
hsa-miR-4485-3p	*GALNT14*	UDP-N-acetyl-alpha-D-galactosamine:polypeptide N-acetylgalactosaminyltransferase 14 (GalNAc-T14)	−0.79
hsa-miR-4485-3p	*AC012215.1*	uncharacterized protein	−0.77
hsa-miR-4485-3p	*PPAPDC3*	phosphatidic acid phosphatase type 2 domain containing 3	−0.75
hsa-miR-4485-3p	*C14orf37*	chromosome 14 open reading frame 37	−0.63
hsa-miR-4485-3p	*RNF41*	ring finger protein 41	−0.62
hsa-miR-4485-3p	*SYP*	synaptophysin	−0.62
hsa-miR-4485-3p	*GPBAR1*	G protein-coupled bile acid receptor 1	−0.62
hsa-miR-4485-3p	*STARD6*	StAR-related lipid transfer (START) domain containing 6	−0.6
hsa-miR-4485-3p	*AZI1*	5-azacytidine induced 1	−0.56
hsa-miR-4485-5p	*AC005003.1*	CDNA FLJ20464 fis, clone KAT06158; HCG1777549; uncharacterized protein	−1
hsa-miR-4485-5p	*CCDC142*	coiled-coil domain containing 142	−0.97
hsa-miR-4485-5p	*RNF165*	ring finger protein 165	−0.9
hsa-miR-4485-5p	*DUSP13*	dual specificity phosphatase 13	−0,9
hsa-miR-4485-5p	*ZNF667*	zinc finger protein 667	−0.89
hsa-miR-4485-5p	*FICD*	FIC domain containing	−0.84
hsa-miR-4485-5p	*TNFSF14*	tumor necrosis factor (ligand) superfamily, member 14	−0.84
hsa-miR-4485-5p	*RRP36*	ribosomal RNA processing 36 homolog (*S. cerevisiae*)	−0.83
hsa-miR-4485-5p	*KLHL35*	kelch-like family member 35	−0.78
hsa-miR-4485-5p	*RP11-94B19.4*	uncharacterized protein	−0.77
hsa-miR-4716	*FN3K*	fructosamine 3 kinase	−2.66
hsa-miR-4716	*KCNC3*	potassium voltage-gated channel, Shaw-related subfamily, member 3	−1.61
hsa-miR-4716	*PDX1*	pancreatic and duodenal homeobox 1	−1.43
hsa-miR-4716	*MSI1*	musashi RNA-binding protein 1	−1.4
hsa-miR-4716	*FAM222B*	family with sequence similarity 222, member B	−1.38
hsa-miR-4716	*MT-ND4L*	mitochondrially encoded NADH dehydrogenase 4L	−1.36
hsa-miR-4716	*SRF*	serum response factor (c-fos serum response element-binding transcription factor)	−1.29
hsa-miR-4716	*PPP2R2D*	protein phosphatase 2, regulatory subunit B, delta	−1.23
hsa-miR-4716	*PDCD6*	programmed cell death 6	−1.17
hsa-miR-4716	*C6orf223*	chromosome 6 open reading frame 223	−1.16
hsa-miR-4722-3p	*ITPKB*	inositol-trisphosphate 3-kinase B	−1.29
hsa-miR-4722-3p	*TLCD2*	TLC domain containing 2	−1.28
hsa-miR-4722-3p	*FAM83F*	family with sequence similarity 83, member F	−1.06
hsa-miR-4722-3p	*TRAF1*	TNF receptor-associated factor 1	−0.94
hsa-miR-4722-3p	*GGT6*	gamma-glutamyltransferase 6	−0.93
hsa-miR-4722-3p	*CLCN2*	chloride channel, voltage-sensitive 2	−0.92
hsa-miR-4722-3p	*SYT12*	synaptotagmin XII	−0.91
hsa-miR-4722-3p	*NFAM1*	NFAT-activating protein with ITAM motif 1	−0.88
hsa-miR-4722-3p	*PRR15L*	proline rich 15-like	−0.87
hsa-miR-4722-3p	*SRL*	sarcalumenin	−0.84
hsa-miR-6723-5p	*GNGT1*	guanine nucleotide-binding protein (G protein), gamma transducing activity polypeptide 1	−0.77
hsa-miR-6723-5p	*CCDC90B*	coiled-coil domain containing 90B	−0.75
hsa-miR-6723-5p	*RNF186*	ring finger protein 186	−0.74
hsa-miR-6723-5p	*PSG2*	pregnancy specific beta-1-glycoprotein 2	−0.7
hsa-miR-6723-5p	*CHIC1*	cysteine-rich hydrophobic domain 1	−0.69
hsa-miR-6723-5p	*IL17A*	interleukin 17A	−0.68
hsa-miR-6723-5p	*PSG11*	pregnancy specific beta-1-glycoprotein 11	−0.64
hsa-miR-6723-5p	*POC1B*	POC1 centriolar protein B	−0.64
hsa-miR-6723-5p	*TMEM114*	transmembrane protein 114	−0.63
hsa-miR-6723-5p	*PSG3*	pregnancy specific beta-1-glycoprotein 3	−0.62
hsa-miR-486-2	*PTEN*	phosphatase and tensin homolog	−1.45
hsa-miR-486-2	*FOXO1*	Forkhead box O1	−1.36
hsa-miR-486-2	*IGF1*	insulin-like growth factor 1	−1.32
hsa-miR-486-2	*CDK2*	cyclin-dependent kinase 2	−1.29
hsa-miR-486-2	*MMP9*	matrix metallopeptidase 9	−1.22
hsa-miR-486-2	*BCL2*	B-cell lymphoma 2	−1.17
hsa-miR-486-2	*AKT1*	AKT serine/threonine kinase 1	−1.15
hsa-miR-486-2	*CCND1*	cyclin D1	−1.12
hsa-miR-486-2	*SMAD4*	SMAD family member 4	−1.08
hsa-miR-486-2	*VEGFA*	vascular endothelial growth factor A	−1.04
hsa-miR-664b-3p	*TTK*	dual specificity protein kinase	−1.41
hsa-miR-664b-3p	*TRIP13*	thyroid hormone receptor interactor	−1.39
hsa-miR-664b-3p	*MCM10*	DNA replication licensing factor	−1.34
hsa-miR-664b-3p	*NUSAP1*	nucleolar spindle associated protein	−1.33
hsa-miR-664b-3p	*CCNA2*	cyclin A2	−1.3
hsa-miR-664b-3p	*BIRC5*	survivin	−1.27
hsa-miR-664b-3p	*CDC25C*	cell division cycle 25C	−1.24
hsa-miR-664b-3p	*TOP2A*	DNA topoisomerase II alpha	−1.23
hsa-miR-664b-3p	*CDCA5*	cell division cycle associated 5	−1.21
hsa-miR-664b-3p	*CENPF*	centromere protein F	−1.19

**Table 6:** Top 10 enriched GO biological processes identified by Enrichr using predicted miRNA target genes.

Name	*p*-Value	Adjusted *p*-Value	Odds Ratio	Combined Score
Spindle Assembly Checkpoint Signaling (GO:0071173)	0.00001379	0.005249	32.60	364.80
Mitotic Spindle Assembly Checkpoint Signaling (GO:0007094)	0.00001379	0.005249	32.60	364.80
Mitotic Spindle Checkpoint Signaling (GO:0071174)	0.00001379	0.005249	32.60	364.80
Negative Regulation of Mitotic Metaphase/Anaphase Transition (GO:0045841)	0.00001850	0.005282	29.98	326.76
Positive Regulation of Mitotic Cell Cycle Phase Transition (GO:1901992)	0.00002705	0.005677	16.01	168.35
Ras Protein Signal Transduction (GO:0007265)	0.00003634	0.005677	14.99	153.20
Mitotic Cell Cycle Phase Transition (GO:0044772)	0.00003782	0.005677	10.67	108.61
Regulation of Mitochondrial Membrane Potential (GO:0051881)	0.00003977	0.005677	24.17	244.94
Negative Regulation of Extrinsic Apoptotic Signaling Pathway (GO:2001237)	0.00004793	0.005679	14.09	140.12
Regulation of G2/M Transition of Mitotic Cell Cycle (GO:0010389)	0.00004974	0.005679	22.71	225.00

## Data Availability

Data are contained within the article.

## Supplementary Materials

The supporting information can be downloaded at: https://www.mdpi.com/article/10.3390/ijms26146623/s1.
