# Supplementary material for: Determination of Differential miRNA Expression Profile in People with Noise-Induced Hearing Loss
Source: Int J Mol Sci. 2025 Jul 10;26(14):6623. doi: 10.3390/ijms26146623 (PMC12294328; doi:10.3390/ijms26146623)
Supplement: Supplementary file 1 [file ijms-26-06623-s001.zip › ijms-3683145-supplementary.pdf]

Supplementary Table 1. Probe-Level Quality Control (QC) Summary for Patients with Comorbidities

Patient ID	pm mean	Background mean	Hybridization Control
Patient 4	123.53	57.70	Pass
Patient 6	86.21	49.64	Pass
Patient 8	120.89	65.82	Pass
Patient 9	145.42	67.85	Pass
Patient 11	127.73	55.89	Pass

This table presents the probe-level QC metrics for the five patients with documented comorbid conditions (Patient IDs 4, 6, 8, 9, and 11). Key metrics include mean probe intensity (pm mean), average background signal, and hybridization control status, confirming that all samples passed the platform's recommended thresholds.

Supplementary Table 2. Predicted Target Genes of Selected miRNAs

**miRNA**	**Number of Predicted Target Genes**	**Example Validated Targets**
hsa-miR-486-2	132	*PTEN, FOXO1, IGF1*
hsa-miR-664b-3p	108	*TOP2A, CCNB1, CDC25A*
hsa-miR-6723-5p	76	*SPARC, E2F1, STAT3*
